# SARS-CoV-2 specific immune responses in overweight and obese COVID-19 patients

**DOI:** 10.3389/fimmu.2023.1287388

**Published:** 2023-11-02

**Authors:** Therese Bredholt Onyango, Fan Zhou, Geir Bredholt, Karl A. Brokstad, Sarah Lartey, Kristin G.-I. Mohn, Türküler Özgümüs, Bård Reiakvam Kittang, Dagrun Waag Linchausen, Shahin Shafiani, Rebecca Elyanow, Bjørn Blomberg, Nina Langeland, Rebecca Jane Cox, Bergen COVID-19 Research Group, Amit Bansal

**Affiliations:** ^1^ Influenza Centre, Department of Clinical Science, University of Bergen, Bergen, Norway; ^2^ Department of Clinical Science, University of Bergen, Bergen, Norway; ^3^ Department of Safety, Chemistry and Biomedical Laboratory Sciences, Western Norway University of Applied Sciences, Bergen, Norway; ^4^ Department of Medicine, Haukeland University Hospital, Bergen, Norway; ^5^ Department of Global Public Health and Primary Care, University of Bergen, Bergen, Norway; ^6^ Department of Medicine, Haraldsplass Deaconess Hospital, Bergen, Norway; ^7^ Bergen Municipality Emergency Clinic, Bergen, Norway; ^8^ Adaptive Biotechnologies, Seattle, WA, United States; ^9^ National Advisory Unit for Tropical Infectious Diseases, Haukeland University Hospital, Bergen, Norway; ^10^ Department of Microbiology, Haukeland University Hospital, Bergen, Norway

**Keywords:** COVID-19, obesity, overweight, spike, neutralising, cellular, TCR, vaccination

## Abstract

Obesity is a known risk factor for severe respiratory tract infections. In this prospective study, we assessed the impact of being obese or overweight on longitudinal SARS-CoV-2 humoral and cellular responses up to 18 months after infection. 274 patients provided blood samples at regular time intervals up to 18 months including obese (BMI ≥30, n=32), overweight (BMI 25-29.9, n=103) and normal body weight (BMI 18.5-24.9, n=134) SARS-CoV-2 patients. We determined SARS-CoV-2 spike-specific IgG, IgA, IgM levels by ELISA and neutralising antibody titres by neutralisation assay. RBD- and spike-specific memory B cells were investigated by ELISpot, spike- and non-spike-specific IFN-γ, IL-2 and IFN-γ/IL-2 secreting T cells by FluoroSpot and T cell receptor (TCR) sequencing was performed. Higher BMI correlated with increased COVID-19 severity. Humoral and cellular responses were stronger in overweight and obese patients than normal weight patients and associated with higher spike-specific IgG binding titres relative to neutralising antibody titres. Linear regression models demonstrated that BMI, age and COVID-19 severity correlated independently with higher SARS-CoV-2 immune responses. We found an increased proportion of unique SARS-CoV-2 specific T cell clonotypes after infection in overweight and obese patients. COVID-19 vaccination boosted humoral and cellular responses irrespective of BMI, although stronger immune boosting was observed in normal weight patients. Overall, our results highlight more severe disease and an over-reactivity of the immune system in overweight and obese patients after SARS-CoV-2 infection, underscoring the importance of recognizing overweight/obese individuals as a risk group for prioritisation for COVID-19 vaccination.

## Introduction

Obesity increases susceptibility to respiratory tract infections and is associated with an elevated risk of developing severe disease. The negative impact of obesity is mediated by various mechanisms, involving direct mechanical effects on ventilation, and physiological alterations, including chronic inflammation and impaired immune responses (1). During the coronavirus disease 2019 (COVID-19) pandemic, body mass index (BMI) has been found to correlate with COVID-19 severity (2–4), with obesity and being overweight emerging as risk factors for severe disease outcomes (5–7).

Adipose tissue stores energy in the form of triglycerides but is also an endocrine organ involved in production and secretion of various cytokines, chemokines, and hormones. Excess amounts of adipose tissue perturb the balance between anti-inflammatory and pro-inflammatory signals, resulting in chronic low-grade inflammation with increased levels of C-reactive protein, interleukin (IL)-6 and tumour necrosis factor alpha (8, 9). The hormone leptin, which is produced in adipose tissue, is increased in obese individuals and has been implicated in attenuated antiviral type I interferon (IFN-I) responses (10). Low IFN-I responses have been reported in association with severe COVID-19 (11, 12). Inefficient or delayed IFN-I responses can result in higher severe acute respiratory coronavirus 2 (SARS-CoV-2) viral loads and may in part explain why obesity represents a risk factor for severe disease (13, 14).

Contradictory results regarding the impact of obesity on immune responses following SARS-CoV-2 infection have been reported with both negative and positive correlations between BMI/obesity and humoral responses (15–17). Obesity is known to have a negative impact on immune responses following vaccination against influenza, tetanus, hepatitis B and rabies. For COVID-19 vaccination, impaired humoral and cellular responses after vaccination in SARS-CoV-2 naïve obese and overweight individuals have been reported (18–21). A large population-based cohort study suggested that COVID-19 vaccination had comparable vaccine effectiveness in overweight and obese vaccinees as normal weight individuals, providing similar levels of protection against severe COVID-19 (22). However, an increased risk of severe COVID-19 outcomes were found for individuals of high and low BMIs.

In our current study we investigated the association between being overweight or obese and longitudinal anti-SARS-CoV-2 humoral and cellular immune responses in a cohort comprising COVID-19 patients diagnosed in Bergen, Norway, during the first pandemic wave (23). Furthermore, our study evaluates the impact of COVID-19 vaccination after recovery from infection in overweight, obese and normal weight individuals.

## Materials and methods

### Study population

COVID-19 patients were recruited prospectively during the first pandemic wave of SARS-CoV-2 in Bergen, Norway, in 2020 from Bergen Municipality Emergency Clinic (BMEC) and the two main city hospitals, Haukeland University Hospital and Haraldsplass Deaconess Hospital. Confirmation of SARS-CoV-2 infection was based on reverse transcription polymerase chain reaction (RT-PCR) of nasopharyngeal specimens or based on the presence of convalescent SARS-CoV-2 specific serum antibodies 2 months after acute COVID-19.

### Ethical considerations

Participants provided written informed consent. For patients unable to provide consent, informed consent was signed by their next-of-kin. COVID-19 survivors subsequently signed informed consent to continue in the study. The study was conducted according to the guidelines of the Declaration of Helsinki and approved by the Regional Committee for Medical and Health Research Ethics in Western Norway (#118664).

### Collection of clinical data and blood samples

Relevant demographic and clinical data were registered in an electronic case report form (eCRF), using the Research Electronic Data Capture tools (REDCap; Vanderbilt University, Nashville, TN, USA). Data included information on gender, age, BMI, comorbidities, COVID-19 symptoms, and COVID-19 vaccination status. Blood sampling started in March 2020 and the final follow-up was 18 months later in October-December 2021. Blood samples were collected at approximately 2, 4, 6, 12 and 18 months after symptom onset.

### Blood collection and processing

Serum was collected from clot activator tubes (CAT, BD, UK), aliquoted and stored at -70°C. Serum was thawed, heat-inactivated (56°C, 1 hour), and batch analysed in serological assays (ELISA, neutralisation assay). Peripheral blood mononuclear cells (PBMCs) were isolated using Cell Preparation Tubes (CPT, BD, UK) according to the manufacturer’s instructions, diluted in cell culture medium (RPMI-1640 with L-glutamine (Lonza), 10% heat-inactivated foetal bovine serum (FBS, Hyclone), 100 U/ml penicillin and 0.1 mg/ml streptomycin (Sigma-Aldrich)) and used directly in memory B cell ELISpot and T cell FluoroSpot assays. Blood for T cell receptor (TCR) sequencing was collected in EDTA tubes (BD, UK), frozen at -70°C, and shipped on dry ice for sequencing by Adaptive Biotechnologies (Seattle, WA, USA).

### Antigens and peptides

The SARS-CoV-2 (Wuhan-Hu-1 isolate) receptor binding domain (RBD) protein and surface glycoprotein (spike) for ELISA were produced in-house from constructs provided by Professor Florian Krammer (24). Libraries of 17-mer synthetic peptides with overlaps of 10 amino acids (> 80% pure) covering the full length of SARS-CoV-2 spike and non-spike (nucleocapsid and matrix proteins) of the USA-WA1/2020 strain were obtained from BEI Resources (VA, USA). Peptides were solubilized in dimethyl sulphoxide (DMSO; ≥ 99.9%), pooled and diluted in medium (final DMSO concentration <0.5%). Peptides for spike were combined in two distinct pools, S1 (amino acid (a.a.) 1-689) and S2 (a.a. 680-1273).

### Enzyme-linked immunosorbent assay

Spike-specific IgG, IgA, and IgM endpoint titres were determined by enzyme-linked immunosorbent assay (ELISA). RBD screening and spike ELISA were performed as previously described (24), with some modifications (25, 26). Serum from a hospitalised COVID-19 patient and the monoclonal antibody CR3022 were used as positive controls (27, 28), whereas pooled pre-pandemic sera (n=128) were used as a negative control (26). Endpoint titres were calculated as the reciprocal of the serum dilution giving an optical density (OD) value of 3 standard deviations above the mean of the negative control. Negative samples were assigned a value of 50, half of the starting dilution of 1/100, for calculation purposes.

### Microneutralisation assay

Neutralising antibody titres were determined by the microneutralisation assay in a certified biosafety level 3 laboratory, as previously described (26). A local SARS-CoV-2 isolate from March 2020 (hCoV-19/Norway/Bergen-01/2020, GISAID accession ID EPI_ISL_541970) was used in this assay. Neutralisation titres were determined as the reciprocal of the serum dilution giving 50% inhibition of virus infectivity (half maximal inhibitory concentration, IC_50_). Negative samples were assigned a value of 10, half of the starting dilution of 1/20, for calculation purposes.

### Memory B cell ELISpot

PBMCs were stimulated at 1x10^6^ cells/ml in medium supplemented with 1 μg/ml R848 (Mabtech AB, Sweden) and 10 ng/ml rhIL-2 (Mabtech), or medium alone (negative control) for 6 days (37°C, 5% CO_2_). ELISpot plates (MultiscreenHTS MSHA N45 10, Millipore) were coated with 10 μg/ml spike protein, 10 μg/ml RBD and 15 μg/ml anti-human IgG (MT91/145, Mabtech) in PBS (Phosphate Buffered Saline), or PBS only (control) at 4°C overnight. Stimulated and non-stimulated PBMCs were transferred in duplicate to ELISpot plates and incubated for 16 hours (37°C, 5% CO_2_). Plates were incubated with 1 µg/ml biotinylated anti-IgG mAbs (MT78/145, Mabtech) and Streptavidin-HRP (1:1000, Mabtech). Spots were developed with 3,3’,5,5’-Tetramethyl-benzidine (TMB) ELISpot substrate (MabTech) and counted using an ELISpot reader (Advanced Imaging Devices, Germany). SARS-CoV-2 spike-specific spots were calculated as the mean of duplicate wells, subtracting spots in negative control wells, and presented as spot forming units (SFU) per 1x10^6^ PMBCs.

### Interferon-gamma and interleukin-2 FluoroSpot assay

Antigen-specific interferon-gamma (IFN-γ), interleukin-2 (IL-2), and double-positive IFN-γ/IL-2 cytokine-secreting T cells were quantified with FluoroSpot kits (Mabtech AB, Sweden), as previously described (29). Average SFU were counted using a fluorescence reader with FITC and Cy3 filters (Advanced Imaging Devices, Germany) and background from negative controls were subtracted.

### SARS-CoV-2 associated T cell receptor β sequences

The immunoSEQ Assay (Adaptive Biotechnologies, Seattle, WA, USA) is a molecular tool for quantification and monitoring of T cell responses to SARS-CoV-2 that is high throughput, sensitive, and that does not rely on live cells (30, 31).

In short, genomic DNA was extracted from blood collected in EDTA tubes using the Qiagen DNeasy Blood Extraction Kit (QIAGEN, Germantown, MD) and amplified in a bias-controlled multiplex PCR (Polymerase Chain Reaction), followed by high-throughput sequencing. SARS-CoV-2 associated CDR3 regions of TCRβ chains were sequenced as previously described (30, 31).

TCRβ sequences were statistically associated with SARS-CoV-2 using case and control repertoires as described in (32). SARS-CoV-2 associated sequences were associated with CD4 and CD8 spike and non-spike antigens using annotated TCRβs from the immunoSEQ T-MAP™ COVID platform. ImmunoSEQ^®^ T-MAP™ COVID is a TCR sequence-based approach to quantitatively assess the T cell response to SARS-CoV-2. This approach utilizes a multiplexed experimental platform to interrogate T cell repertoires with large numbers of query antigens to identify SARS-CoV-2-specific TCRs in the context of HLA (33).

### Statistical analysis

GraphPad Prism (version 9.5.1; La Jolla, CA, USA) was used to analyse data and generate figures. Antibody titres were log-transformed and compared between groups and time-points, using an unpaired, non-parametric Kruskal-Wallis test with Dunn’s multiple comparisons test to evaluate statistical significance. Correlation was evaluated by computing non-parametric Spearman correlation with a two-tailed 95% confidence interval (CI).

R version 4.3.0 (R Foundation for Statistical Computing, Vienna, Austria) was used to generate linear regression models where variables were adjusted for BMI (continuous), age (continuous), gender (reference: male), any comorbidity (reference: no comorbidity) and COVID-19 severity (category). Study participants with BMI<18.5 were excluded in the models. Results are presented as adjusted estimates, 95% CIs and p-values. Log-transformed values of dependent variables were used in the models. Exponentiated values of estimate (geometric mean) and CI are presented in the tables. For TCR breadth measurements, a small number (10^-5^) was added to every value to handle zero values of measurements, which are problematic for log-transformation. For TCR depth measurements, values were shifted on the original scale to have all positive values, similarly, to handle the problem with log-transformation.

## Results

### Study population

The study population included 274 patients from the first COVID-19 wave comprising both home-isolated patients, with asymptomatic (n=3) and moderate disease (n=210), and hospitalised patients, with severe (n=52) to critical disease (n=9). The cohort was divided into groups based on BMI consisting of 5 underweight (BMI<18.5 kg/m^2^), 134 normal weight (BMI=18.5-24.9 kg/m^2^, median age 45 years, 41% male), 103 overweight (BMI=25-29.9 kg/m^2^, median age 52 years, 58% male) and 32 obese (BMI≥30 kg/m^2^, median age 53 years, 59% male) COVID-19 patients (**Table 1**). Forty percent (40%) of normal weight participants reported comorbidities, whereas the percentages increased to 49% for overweight and 66% for obese patients. Hypertension was associated with increasing body weight.

**Table 1:** Baseline demographic and clinical data.

	Underweight <18.5 kg/m^2^	Normal weight 18.5-24.9 kg/m^2^	Overweight 25-29.9 kg/m^2^	Obese ≥30 kg/m^2^
N = 274				
n (%)	5 (2%)	134 (49%)	103 (38%)	32 (12%)
Gender				
Male: n (%)	0 (0%)	55 (41%)	60 (58%)	19 (59%)
Female: n (%)	5 (100%)	79 (59%)	43 (42%)	13 (41%)
Age: range, median	16-64, 22	16-84, 45	21-80, 52	16-78, 53
Hospitalised: n (%)	0 (0%)	19 (14%)	26 (25%)	16 (50%)
Home-isolated: n (%)	5 (100%)	115 (86%)	77 (75%)	16 (50%)
Severity score*:				
1 asymptomatic	1 (20%)	1 (<1%)	1 (1%)	0
2 home-isolated	4 (80%)	114 (85%)	76 (74%)	16 (50%)
4 hospitalised with medical needs	0 (0%)	14 (10%)	10 (10%)	5 (16%)
5 hospitalised needing oxygen	0 (0%)	5 (4%)	12 (12%)	6 (19%)
6 hospitalised needing ventilation	0 (0%)	0 (0%)	2 (2%)	1 (3%)
7 hospitalised needing respirator	0 (0%)	0 (0%)	2 (2%)	4 (13%)
Comorbidities:				
Comorbidity (%)	3 (60%)	54 (40%)	50 (49%)	21 (66%)
Any lung disease (%)	1 (20%)	16 (12%)	14 (14%)	4 (13%)
Chronic heart disease (%)	0 (0%)	4 (3%)	14 (14%)	3 (9%)
Hypertension (%)	0 (0%)	7 (5%)	18 (17%)	8 (25%)
Immunosuppressed (%)	0 (0%)	6 (4%)	3 (3%)	1 (3%)

*Severity score was modified after (34).

Study participants provided at least 3 follow-up blood samples at approximately 2, 4, 6, 12 and 18 months post symptom onset to evaluate the kinetics and durability of the humoral and cellular SARS-CoV-2 specific immune responses (**Supplementary Figure 1**).

### Increasing BMI associated with higher risk of severe COVID-19 and hospitalisation

**Figure 1:**
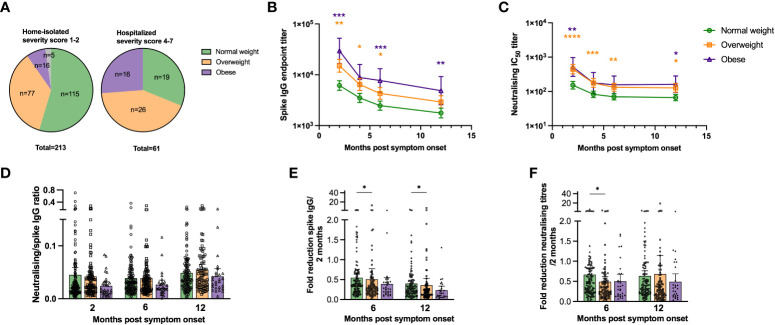
SARS-CoV-2 specific antibody titres are higher in overweight and obese patients. The pie charts in **(A)** show the proportion of underweight, normal weight, overweight and obese study participants that were home-isolated (disease severity score 1-2) and hospitalised (disease severity score 4-7). Spike-specific IgG endpoint titres were determined by ELISA **(B)** and neutralising antibody titres (Inhibitory Concentration 50, IC_50_) against SARS-CoV-2 (Wuhan) were determined by neutralisation assay **(C)** at 2, 4, 6 and 12 months post symptom onset for normal weight, overweight and obese patients. The ratio of neutralising to spike-specific IgG levels for normal weigh, overweight and obese patients were calculated for paired samples at 2, 6 and 12 months post symptom onset **(D)**. Fold reduction of spike-specific IgG **(E)** and neutralisation titres **(F)** relative to the 2 months values were determined at 6 and 12 months post symptom onset for normal weight, overweight and obese patients. Patients that were vaccinated against COVID-19 before the 12 months follow-up were excluded from the figures at the 12 months time-point (n=20). The results in **(D–F)** are displayed as means with 95% CIs, the results in **(B, C)** are displayed as geometric means with 95% CIs. In **(B, C)**, statistically significant differences between overweight and normal weight patients are indicated with orange asterisks, and significant differences between obese and normal weight patients indicated with purple asterisks. A non-parametric Kruskal-Wallis test with Dunn’s multiple comparisons test was used to evaluate statistical significance, with * = p<0.05, ** = p<0.01, *** = p<0.001 and **** = p<0.0001.

Patients were assigned a severity of disease score [modified from (34)] from 1 (asymptomatic) to 7 (hospitalised needing respirator) (**Table 1**). All underweight (n=5, 100%) and 86% of normal weight patients (n=115) were home-isolated with asymptomatic to mild disease (severity scores 1 or 2, respectively), while 14% of normal weight patients (n=19) were hospitalised with moderately severe disease (severity score 4 or 5) (**Figure 1A**). Of the overweight group (n=103), 75% (n=77) were home-isolated, while 25% (n=26) were admitted to hospital (severity score 4-7). Half (n=16) of the obese group (n=32) were home-isolated, while half (n=16) were hospitalised (severity score 4-7), with the highest percentage of respirator patients. In agreement with previous findings (2–4), increasing BMI in our cohort correlated significantly with progression to severe COVID-19 (r=0.2733, p<0.0001).

### Higher spike-specific and neutralising antibody titres in overweight and obese patients

Spike-specific IgG titres were significantly higher in overweight compared to normal weight patients up to 6 months, and in obese compared to normal weight patients up to 12 months (except 4 months) (**Figure 1B**). The lack of significance for the obese group at 4 months is likely due to the relatively small number of obese individuals in the study population. A higher proportion of overweight and obese patients had detectable RBD-specific IgA and IgM antibodies at 2 months than normal weight patients. However, if detected, spike-specific IgA and IgM endpoint titres in the RBD-positive samples were comparable between normal weight, overweight and obese patients (**Supplementary Figure 2**). Linear regression models adjusted for age, gender, any comorbidity and COVID-19 severity, showed that BMI significantly impacted spike-specific IgG titres at 2 months post symptom onset (**Table 2**). The adjusted estimates showed that both age and COVID-19 severity significantly affected IgG titres at all time-points.

**Table 2:** Variables associated with spike IgG and T cell responses after SARS-CoV-2 infection.

Time post infection (months):	Variables:	Spike IgG titer Adjusted estimate (95% CI)	p-value	Spike TCR breadth Adjusted estimate (95% CI)	p-value	Spike TCR depth Adjusted estimate (95% CI)	p-value
**2**	BMI (cont.)	**1.07 (1.03-1.11)**	**0.001**	1.02 (0.99-1.04)	0.151	**1.03 (1.00-1.06)**	**0.024**
	Age (cont.)	**1.02 (1.01-1.03)**	**<0.001**	**1.01 (1.01-1.02)**	**<0.001**	1.00 (1.00-1.01)	0.200
	Gender (ref:male)	0.86 (0.66-1.14)	0.297	0.92 (0.76-1.11)	0.370	1.13 (0.93-1.37)	0.212
	Any comorbidity (ref:no)	1.09 (0.82-1.44)	0.567	1.06 (0.87-1.28)	0.567	0.95 (0.78-1.15)	0.577
	COVID-19 severity (cat.)	**1.68 (1.49-1.90)**	**<0.001**	1.05 (0.96-1.14)	0.279	1.01 (0.93-1.10)	0.818
**6**	BMI (cont.)	1.03 (1.00-1.07)	0.066	**1.04 (1.02-1.06)**	**0.001**	**1.04 (1.01-1.06)**	**0.003**
	Age (cont.)	**1.02 (1.02-1.03)**	**<0.001**	**1.01 (1.01-1.02)**	**<0.001**	**1.01 (1.00-1.01)**	**0.007**
	Gender (ref:male)	1.04 (0.81-1.35)	0.759	1.14 (0.97-1.33)	0.104	**1.31 (1.10-1.57)**	**0.003**
	Any comorbidity (ref:no)	1.12 (0.86-1.46)	0.411	0.99 (0.84-1.16)	0.912	1.02 (0.85-1.23)	0.832
	COVID-19 severity (cat.)	**1.63 (1.45-1.83)**	**<0.001**	**1.10 (1.03-1.18)**	**0.007**	1.04 (0.96-1.13)	0.298
**12**	BMI (cont.)	1.04 (1.00-1.08)	0.073	**1.05 (1.03-1.07)**	**<0.001**	**1.06 (1.03-1.09)**	**<0.001**
	Age (cont.)	**1.02 (1.01-1.03)**	**<0.001**	**1.01 (1.01-1.02)**	**<0.001**	**1.01 (1.00-1.02)**	**0.001**
	Gender (ref:male)	1.28 (0.96-1.70)	0.089	1.05 (0.91-1.22)	0.494	1.19 (0.99-1.43)	0.064
	Any comorbidity (ref:no)	1.12 (0.84-1.51)	0.439	1.07 (0.92-1.24)	0.377	1.03 (0.85-1.24)	0.766
	COVID-19 severity (cat.)	**1.60 (1.41-1.81)**	**<0.001**	1.03 (0.97-1.10)	0.340	1.02 (0.94-1.10)	0.665

Statistically significant results are written in bold font.

Neutralising titres were significantly higher in overweight than normal weight patients up to 12 months, and at 2 (p<0.01) and 12 months (p<0.05) in obese patients (**Figure 1C**). Furthermore, adjusted models demonstrated that BMI, age and COVID-19 severity were independently and significantly associated with neutralising antibody titres (**Supplementary Table 1**). The ratio of neutralising to total SARS-CoV-2 specific antibodies has been suggested to predict COVID-19 severity and survival (35). We calculated the ratio of neutralising to spike-specific IgG titres and found a trend of lower ratios in obese than in overweight and normal weight patients (**Figure 1D**).

We calculated the fold reduction in spike-specific IgG and neutralising titres at 6 and 12 months relative to 2 months post symptom onset (**Figures 1E, F**). The fold reduction was significantly higher for the overweight group compared to the normal weight group for IgG at 6 and 12 months (p<0.05), and for neutralising antibodies at 6 months (p<0.05). Possible SARS-CoV-2 re-infection was observed in 1%-4% of patients at 6 and 12 months by seroconversion of spike IgG and neutralising antibodies, respectively. However, none of the participants tested positive for SARS-CoV-2 during this period.

### Higher spike-specific cellular responses in overweight and obese patients

We analysed the spike- and non-spike (i.e. internal proteins) specific T cell receptor (TCR) repertoire up to 12 months post symptom onset, using the immunoSEQ Assay T-MAP™ COVID platform, a high throughput sequence-based method for quantification of SARS-CoV-2-specific T cell responses (32). We calculated TCR breadth defined as the proportion of unique SARS-CoV-2-specific T cell clonotypes relative to the total TCR repertoire, and TCR depth defined as the extent of SARS-CoV-2-specific T cells expansion. Clonal breadth and depth are calculated as described in (32).

**Figure 2:**
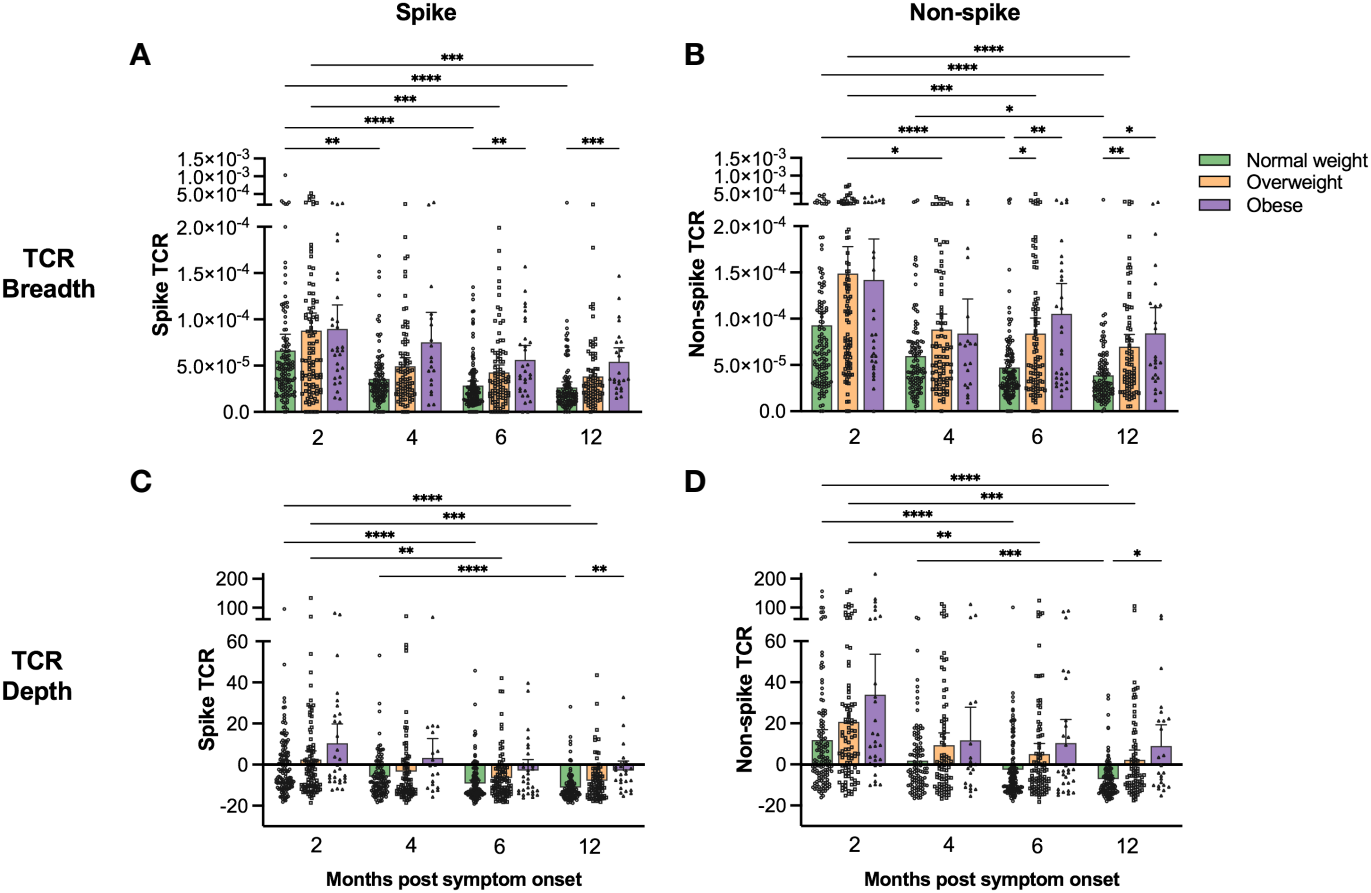
Spike- and non-spike-specific T cell receptor sequencing showed higher clonal breadth and depth in overweight and obese than in normal weight COVID-19 patients. Spike-specific **(A, C)** and non-spike-specific **(B, D)** T cell receptor clonal breadth **(A, B)** and depth **(C, D)** were measured using the immunoSEQ T-MAP™ COVID platform at 2, 4, 6 and 12 months post symptom onset. The results are presented as means with 95% CIs. Patients that were vaccinated against COVID-19 before the 12 months follow-up were excluded from the figures at the 12 months time-point (n=20). A non-parametric Kruskal-Wallis test with Dunn’s multiple comparisons test was used to evaluate statistical significance, with * = p<0.05, ** = p<0.01, *** = p<0.001 and **** = p<0.0001.

Spike and non-spike TCR breadth and depth were highest at 2 months for all patients (**Figures 2A–D**). Generally, overweight and obese patients had higher spike and non-spike specific TCR breadth and depth than the normal weight group, with the differences becoming more pronounced by 6 and 12 months post symptom onset. Linear regression models (adjusted for BMI, age, gender, any comorbidity, and COVID-19 severity) demonstrated that spike and non-spike TCR breadth were significantly associated with age at 2, 6 and 12 months, although BMI and COVID-19 severity were also important at 6 and 12 months (only BMI for spike TCR breadth) (**Table 2** and **Supplementary Table 2**). Spike TCR depth was significantly associated with BMI at 2, 6 and 12 months, whilst age and gender were also important at 6 months, and age at 12 months (**Table 2**). BMI, age and COVID-19 severity were significantly associated with non-spike TCR depth at 6 and 12 months (**Supplementary Table 2**).

**Figure 3:**
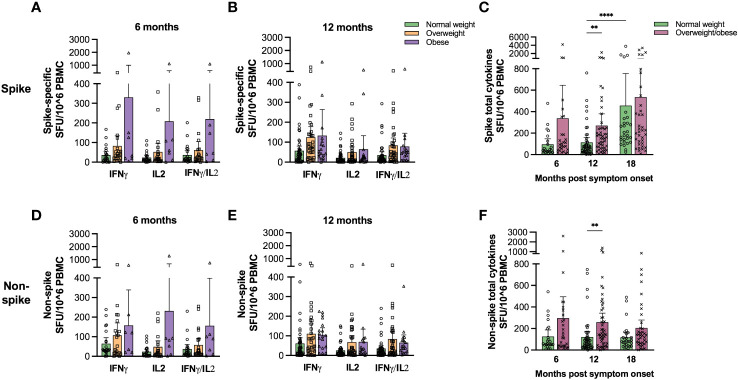
Spike- and non-spike-specific cytokine-secreting T cell frequencies were higher in overweight/obese COVID-19 patients. Frequencies of IFNγ, IL-2 and IFNγ/IL-2 spike- **(A, B)** and non-spike **(D, E)** specific T cells were determined at 6 **(A, D)** and 12 months **(B, E)** for normal weight, overweight and obese COVID-19 patients using FluroSpot assay. The total frequencies of cytokine-secreting spike- **(C)** and non-spike **(F)** specific T cells were determined for normal weight and the combined group of overweight and obese patients 6 and 12 months. Results for participants that were vaccinated against COVID-19 before 12 months (n=9) were excluded from the 12 months time-point. The results following vaccination at the 18 months follow-up are included in **(C, D)**. Results are presented as means with 95% CIs. A non-parametric Kruskal-Wallis test with Dunn’s multiple comparisons test was used to evaluate statistical significance, with ** = p<0.01 and **** = p<0.0001.

To confirm T cell functionality, we assessed the frequencies of spike- and non-spike-specific T cells secreting IFN-γ, IL-2 and IFN-γ/IL-2 at 6 and 12 months by FluoroSpot assay in a subgroup of the study population providing PBMCs (**Supplementary Figure 1**). Generally, we found that the frequencies of cytokine-secreting T cells were higher in overweight and obese than in normal weight patients, although not significantly, possibly due to the relatively small sample size (**Figures 3A, B, D, E**; linear regression models in **Supplementary Table 3**). We therefore combined the groups of overweight and obese patients and found that the frequencies of spike- and non-spike-specific total cytokine-secreting T cells were significantly higher for overweight/obese patients than in normal weight patients at 12 months (**Figures 3C, F**, p<0.01). The frequencies of spike-specific total cytokine-secreting T cells were significantly associated with BMI and age at 12 months in linear regression models, while there was no significant association between BMI and non-spike-specific total cytokine-secreting T cells (**Supplementary Table 4**).

### COVID-19 vaccination significantly boosted humoral and cellular responses

**Figure 4:**
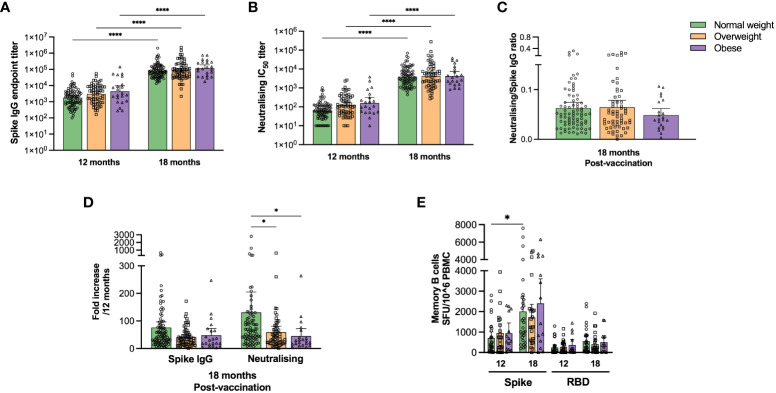
COVID-19 vaccination boosted SARS-COV-2 specific antibody responses in normal weight, overweight and obese individuals. Spike-specific IgG titres were determined by ELISA **(A)** and neutralising antibody titres (IC_50_) by microneutralisation assay **(B)** for paired samples at 12 (pre-vaccination) and 18 months (post-vaccination) for normal weight, overweight and obese patients. The ratios of neutralising/spike-specific IgG titres were calculated post-vaccination at 18 months **(C)**. The fold increase in spike-specific IgG and neutralising titres post-vaccination was calculated relative to the respective values at 12 months **(D)**. Results from study participants that had received COVID-19 vaccine before 12 months (n=20), and unvaccinated (n=11) or unknown vaccination status (n=1) at 18 months were excluded. Frequencies of spike- and RBD-specific IgG MBCs were assessed in 65 patients with paired samples by ELISpot assay at 12 and 18 months. Participants were excluded from the analyses if they were COVID-19 vaccinated before 12 months (n=6) or were unvaccinated at 18 months (n=1) **(D)**. The results in **(A, B)** are presented as geometric means with 95% CIs, the results in **(C–E)** are presented as means with 95% CIs. A non-parametric Kruskal-Wallis test with Dunn’s multiple comparisons test was used to evaluate statistical significance, with * = p<0.05 and **** = p<0.0001.

The final follow-up was 18 months after initial SARS-CoV-2 symptom onset, and after the implementation of the COVID-19 vaccination campaign. Ninety-three normal weight, 78 overweight and 27 obese participants attended the final follow-up of whom 186 (94%) had received COVID-19 vaccination and 169 had paired samples (**Figure 4** and **Supplementary Table 5**).

Vaccination significantly boosted spike-specific IgG and neutralising antibody titres, regardless of body weight category (**Figures 4A, B**, p<0.0001), although no significant differences were observed between the weight categories or in functional neutralising to spike-specific IgG ratios (**Figure 4C**). We also found a significant fold increase of neutralising antibodies in normal weight compared to overweight and obese patients (**Figure 4D**, p<0.05).

Spike- and RBD-specific IgG MBC responses after vaccination were further assessed in 65 individuals with paired samples by ELISpot assay (**Figure 4E**). COVID-19 vaccination significantly (p<0.05) boosted spike-specific MBCs only in normal weight patients. Furthermore, spike-specific total cytokine-secreting T cells increased significantly (p<0.0001) after vaccination only in normal weight, but not in overweight/obese participants (**Figure 3C**). The main findings of this study (**Figures 1** **–** **4**) are summarized in **Supplementary Table 6**.

## Discussion

Obesity is a known risk factor for severe COVID-19, and being overweight has also been associated with more severe illness (5–7). Furthermore, severe COVID-19 is characterized by an increased aberrant immune response (11, 35, 36). Here, we conducted a longitudinal study to investigate immune responses and immune memory after COVID-19 infection and subsequent vaccination, focusing on the impact of being overweight and obese. Our findings demonstrate a correlation between increasing BMI and severe COVID-19, as well as significantly higher levels of spike-specific IgG and neutralising antibody titres up to twelve months post infection in overweight and obese patients compared to normal weight patients, agreeing with previous reports (16, 17). BMI independently and significantly impacted spike-specific IgG and neutralising titres at two months post symptom onset, supporting reports of a positive association between antibody levels and obesity (16, 17). Previous findings showed that BMI and age were independently associated with higher SARS-CoV-2 antibody levels in convalescent COVID-19 patients (17), while our present data show that BMI and age impact both spike-specific IgG and neutralising antibodies as well as durable spike and non-spike TCR breadth and depth. This indicates an increased proportion of unique SARS-CoV-2 specific T cell clonotypes that rapidly expanded and were maintained after infection, suggesting an over-reactivity of the immune system in overweight and obese patients.

More than 90% of neutralising antibodies have been shown to bind the RBD of the spike protein (37), with neutralisation associated with IgG specific for conformational spike and RBD epitopes (38). The ratio of neutralising to total SARS-CoV-2 specific antibodies was found to be lower in severe COVID-19 (35). Severe and fatal COVID-19 cases had delayed and reduced production of neutralising antibodies (39, 40). Here we found a trend of lower ratios of neutralising to spike IgG titres in obese relative to overweight and normal weight individuals following both initial infection and vaccination, highlighting obese individuals as a risk group for vaccine prioritisation. In agreement, reduced levels of neutralising antibodies have been detected in obese individuals following COVID-19 vaccination (18, 20).

Higher frequencies of SARS-CoV-2 specific memory T cells have been found in severe COVID-19 (29, 41, 42). Convalescent critically ill patients were shown to have persisting SARS-CoV-2 specific T cell responses for more than one year after discharge from hospital, with the magnitude being associated with the length of hospital stay (43). Our overweight/obese patients had higher frequencies of spike- and non-spike-specific T cells than normal weight COVID-19 patients at twelve months. Furthermore, we found that spike and non-spike TCR breadth and depth were significantly higher for overweight and obese compared to normal weight COVID-19 patients, although the patients age and disease severity were also important. TCR sequencing has been reported to correlate with COVID-19 disease severity, as well as with SARS-CoV-2 neutralising antibody titres (44). Interestingly, we have previously shown that long COVID (post COVID-19 condition) correlated with higher levels of spike-specific IgG (23) and spike-specific CD4+ TCR depth (45), indicating that increasing BMI could be associated with persisting symptoms. In agreement with this, an increased risk for long COVID has been found for overweight/obese patients (46).

We found that RBD- and spike-specific MBCs were maintained in most of our convalescents, regardless of weight category, at twelve months, although often at higher frequencies in overweight/obese patients. This is in agreement with previous reports showing maintenance of MBCs for up to fifteen months post infection (47, 48). Based on the correlation between BMI and severity, our observations support the findings that the maintenance and the magnitude of RBD MBCs correlate with disease severity after twelve months (49).

Hybrid immunity, the combination of natural infection and vaccination, induces a broader and more durable immune response than vaccination alone (48, 50). Here, we observed that vaccination after infection induced a significant increase in spike-specific IgG and neutralising antibodies in all weight groups. Cellular immune responses were boosted by vaccination, with significant increases in spike-specific MBC frequencies and total spike-specific T cells for normal weight patients. The trend of a lower ratio of neutralising to spike IgG for obese patients could indicate a reduced neutralising potency in this group. However, protective immunity relies on both cellular and humoral responses, and the induction of SARS-CoV-2 specific T cells has been suggested to have a central role, with the potential to provide cross-reactive protection against new viral variants and with a slower decay compared to neutralising antibodies (51–54).

The strengths of this study include that it comprises an almost complete cohort from the first pandemic wave of COVID-19 in Bergen, Norway. Our study had good follow-up rates reducing bias, and both humoral and cellular responses were studied in detail. Further strengths are the stratified analysis of confounding factors such as age and severity in multivariable models. Caveats of the study include that the Norwegian population has relatively low levels of obesity, being reflected in the relatively few obese individuals in the study population. Furthermore, the study was conducted with Wuhan virus infected patients and there may be differences with other SARS-CoV-2 variants.

In summary, our finding supports the concept that over-reactivity of the immune system, and possibly autoimmune effects of antibodies could play a role in the pathogenesis of severe COVID-19. We showed that increasing BMI is correlated with severe disease and associated with increased humoral and cellular responses up to twelve months following SARS-CoV-2 infection, with lower levels of neutralising to spike IgG antibodies in the obese group following infection and vaccination. Vaccination boosted humoral and cellular SARS-CoV-2 immune responses to similar levels in normal weight, overweight and obese convalescent COVID-19 patients, highlighting the importance of overweight/obese individuals as a risk group for prioritisation for COVID-19 vaccination.

## Data availability statement

The raw data supporting the conclusions of this article will be made available by the authors, without undue reservation.

## Ethics statement

The studies involving humans were approved by Regional Committee for Medical and Health Research Ethics in Western Norway (#118664). The studies were conducted in accordance with the local legislation and institutional requirements. The participants provided their written informed consent to participate in this study.

## Author contributions

TBO: Writing – original draft, Data curation, Formal Analysis. FZ: Data curation, Formal Analysis, Writing – review & editing. GB: Data curation, Formal Analysis, Writing – review & editing. KAB: Writing – review & editing, Project administration. SL: Project administration, Writing – review & editing, Formal Analysis. KG-IM: Writing - review & editing, Project administration. TÖ: Formal Analysis, Writing – review & editing. BRK: Project administration, Writing – review & editing. DWL: Project administration, Writing – review & editing. SS: Formal Analysis, Methodology, Writing – review & editing. RE: Formal Analysis, Methodology, Writing – review & editing. BB: Writing – original draft, Conceptualization, Project administration. NL: Conceptualization, Project administration, Writing – original draft. RC: Conceptualization, Project administration, Writing – original draft.

## COVID-19 research group

Amit Bansal, Anders Madsen, Camilla Tøndel, Elisabeth Berg Fjelltveit, Hanne Søyland, Helene Heitmann Sandnes, Jan Stefan Olofsson, Juha Vahokoski, Kristin Risa, Lena Hansen, Mai-Chi Trieu, Marianne Sævik and Nina Urke Ertesvåg.
