# Supplementary material for: SARS-CoV-2 specific immune responses in overweight and obese COVID-19 patients
Source: Front Immunol. 2023 Nov 2;14:1287388. doi: 10.3389/fimmu.2023.1287388 (PMC10653322; doi:10.3389/fimmu.2023.1287388)
Supplement: Supplementary file 1 [file Table_1.docx]

**Supplementary table 1**

Variables associated with neutralising antibody levels after SARS-CoV-2 infection

| **Time post infection (months):** | **Variables:** | **Neutralising (IC_50_) titer**  **Adjusted estimate (95% CI) p-value** | |
| --- | --- | --- | --- |
| **2** | BMI (cont.) | **1.06 (1.01-1.11)**  **1.02 (1.01-1.04)** | **0.011** |
|  | Age (cont.) |  | **<0.001** |
|  | Gender (ref:male) | **0.70 (0.50-0.98)** | **0.039** |
|  | Any comorbidity (ref:no) | 0.99 (0.70-1.41) | 0.970 |
|  | COVID-19 severity (cat.) | **1.58 (1.36-1.83)** | **<0.001** |
| **6** | BMI (cont.) | **1.05 (1.01-1.09**) | **0.015** |
|  | Age (cont.) | **1.02 (1.01-1.03)** | **<0.001** |
|  | Gender (ref:male) | 1.08 (0.80-1.44) | 0.622 |
|  | Any comorbidity (ref:no) | 1.08 (0.80-1.46) | 0.625 |
|  | COVID-19 severity (cat.) | **1.39 (1.22-1.58)** | **<0.001** |
| **12** | BMI (cont.) | **1.05 (1.01-1.10)** | **0.007** |
|  | Age (cont.) | **1.02 (1.01-1.03)** | **<0.001** |
|  | Gender (ref:male) | 1.28 (0.95-1.71) | 0.102 |
|  | Any comorbidity (ref:no) | 1.02 (0.76-1.38) | 0.881 |
|  | COVID-19 severity (cat.) | **1.40 (1.24-1.59)** | **<0.001** |

Statistically significant results are written in bold font.
