# Supplementary material for: SARS-CoV-2 specific immune responses in overweight and obese COVID-19 patients
Source: Front Immunol. 2023 Nov 2;14:1287388. doi: 10.3389/fimmu.2023.1287388 (PMC10653322; doi:10.3389/fimmu.2023.1287388)
Supplement: Supplementary file 2 [file Table_2.docx]

**Supplementary table 2**

Variables associated with non-spike TCR breadth and depth after SARS-CoV-2 infection

| **Time post infection (months):** | **Variables:** | **Non-spike TCR breadth**  **Adjusted estimate (95% CI) p-value** | | **Non-spike TCR depth**  **Adjusted estimate (95% CI) p-value** | |
| --- | --- | --- | --- | --- | --- |
| **2** | BMI (cont.) | 1.02 (1.00-1.05) | 0.074 | **1.03 (1.00-1.06)** | **0.048** |
|  | Age (cont.) | **1.01 (1.01-1.02)** | **<0.001** | 1.01 (1.00-1.01) | 0.086 |
|  | Gender (ref:male) | 0.91 (0.75-1.10) | 0.329 | 0.99 (0.81-1.21) | 0.910 |
|  | Any comorbidity (ref:no) | 0.94 (0.77-1.15) | 0.568 | 0.89 (0.72-1.09) | 0.256 |
|  | COVID-19 severity (cat.) | 1.09 (0.99-1.19) | 0.074 | 1.08 (0.98-1.18) | 0.110 |
| **6** | BMI (cont.) | **1.04 (1.02-1.06)** | **0.001** | **1.03 (1.00-1.06)** | **0.030** |
|  | Age (cont.) | **1.01 (1.01-1.02)** | **<0.001** | **1.01 (1.00-1.01)** | **0.006** |
|  | Gender (ref:male) | 0.96 (0.81-1.13) | 0.620 | 1.14 (0.94-1.39) | 0.172 |
|  | Any comorbidity (ref:no) | 1.00 (0.85-1.19) | 0.958 | 0.95 (0.78-1.16) | 0.599 |
|  | COVID-19 severity (cat.) | **1.12 (1.04-1.20)** | **0.004** | **1.11 (1.02-1.21)** | **0.016** |
| **12** | BMI (cont.) | **1.04 (1.02-1.07)** | **<0.001** | **1.05 (1.03-1.08)** | **<0.001** |
|  | Age (cont.) | **1.01 (1.01-1.02)** | **<0.001** | **1.01 (1.00-1.02)** | **0.002** |
|  | Gender (ref:male) | 0.99 (0.85-1.15) | 0.891 | 1.11 (0.92-1.35) | 0.267 |
|  | Any comorbidity (ref:no) | 1.01 (0.86-1.18) | 0.899 | 0.98 (0.81-1.20) | 0.872 |
|  | COVID-19 severity (cat.) | **1.10 (1.03-1.18)** | **0.006** | **1.13 (1.04-1.23)** | **0.005** |

Statistically significant results are written in bold font.
