# Supplementary material for: SARS-CoV-2 specific immune responses in overweight and obese COVID-19 patients
Source: Front Immunol. 2023 Nov 2;14:1287388. doi: 10.3389/fimmu.2023.1287388 (PMC10653322; doi:10.3389/fimmu.2023.1287388)
Supplement: Supplementary file 3 [file Table_3.docx]

**Supplementary table 3**

Variables associated with frequencies of spike- and non-spike-specific cytokine secreting T cells after SARS-CoV-2 infection

| **Time post infection (months):** | **Variables:** | **Spike-specific IL-2**  **SFU/10^6^ PBMC**  **Adjusted estimate (95% CI) p-value** | | **Spike-specific IFN-γ**  **SFU/10^6^ PBMC**  **Adjusted estimate (95% CI) p-value** | | **Spike-specific IFN-γ/IL-2**  **SFU/10^6^ PBMC**  **Adjusted estimate (95% CI) p-value** | |
| --- | --- | --- | --- | --- | --- | --- | --- |
| **6** | BMI (cont.) | 1.03 (0.70-1.50) | 0.891 | 1.37 (0.89-2.12) | 0.147 | 1.21 (0.89-1.64) | 0.223 |
|  | Age (cont.) | 0.96 (0.88-1.05) | 0.351 | 0.91 (0.82-1.01) | 0.064 | 1.01 (0.93-1.08) | 0.874 |
|  | Gender (ref:male) | 4.20 (0.30-59.32) | 0.280 | 6.83 (0.33-143.12) | 0.210 | 7.63 (0.90-64.72) | 0.062 |
|  | Any comorbidity (ref:no) | **43.91 (2.58-747.88)** | **0.010** | **77.46 (2.98-2014.61)** | **0.010** | 2.12 (0.22-20.96) | 0.511 |
|  | COVID-19 severity (cat.) | 1.77 (0.67-4.65) | 0.241 | 0.80 (0.26-2.43) | 0.686 | 1.30 (0.59-2.83) | 0.506 |
| **12** | BMI (cont.) | 1.15 (0.92-1.45) | 0.217 | 1.18 (0.95-1.46) | 0.133 | 1.18 (0.99-1.42) | 0.063 |
|  | Age (cont.) | **1.08 (1.01-1.15)** | **0.021** | 1.00 (0.95-1.07) | 0.884 | 1.03 (0.98-1.08) | 0.295 |
|  | Gender (ref:male) | **12.75 (1.72-94.70)** | **0.013** | 2.59 (0.40-16.71) | 0.313 | 4.01 (0.85-19.01) | 0.080 |
|  | Any comorbidity (ref:no) | 1.30 (0.17-9.80) | 0.797 | 1.36 (0.21-8.90) | 0.745 | 0.62 (0.13-2.99) | 0.549 |
|  | COVID-19 severity (cat.) | 1.52 (0.71-3.22) | 0.276 | 1.34 (0.66-2.69) | 0.415 | 1.37 (0.76-2.47) | 0.285 |
| **Time post infection (months):** | **Variables:** | **Non-spike-specific IL-2**  **SFU/10^6^ PBMC**  **Adjusted estimate (95% CI) p-value** | | **Non-spike-specific IFN-γ**  **SFU/10^6^ PBMC**  **Adjusted estimate (95% CI) p-value** | | **Non-spike-specific IFN-γ/IL-2**  **SFU/10^6^ PBMC**  **Adjusted estimate (95% CI) p-value** | |
| **6** | BMI (cont.) | 1.30 (0.88-1.92) | 0.188 | 1.17 (0.79-1.73) | 0.435 | 1.16 (0.93-1.45) | 0.178 |
|  | Age (cont.) | 0.99 (0.90-1.09) | 0.862 | 0.98 (0.89-1.08) | 0.665 | 0.97 (0.92-1.02) | 0.194 |
|  | Gender (ref:male) | 7.55 (0.48-118.59) | 0.146 | 1.02 (0.06-16.35) | 0.989 | 4.51 (0.95-21.31) | 0.057 |
|  | Any comorbidity (ref:no) | 6.82 (0.36-130.31) | 0.196 | 2.02 (0.10-39.50) | 0.635 | **6.59 (1.25-34.75)** | **0.027** |
|  | COVID-19 severity (cat.) | 1.50 (0.55-4.10) | 0.422 | 0.87 (0.31-2.39) | 0.778 | 1.10 (0.62-1.94) | 0.741 |
| **12** | BMI (cont.) | 1.06 (0.86-1.32) | 0.567 | **1.30 (1.04-1.62)** | **0.023** | **1.27 (1.02-1.57)** | **0.031** |
|  | Age (cont.) | 1.01 (0.95-1.08) | 0.642 | 0.99 (0.93-1.05) | 0.689 | 1.05 (0.99-1.12) | 0.095 |
|  | Gender (ref:male) | 3.57 (0.54-23.52) | 0.184 | 2.32 (0.33-16.40) | 0.394 | **13.91 (2.13-90.78)** | **0.006** |
|  | Any comorbidity (ref:no) | 0.52 (0.08-3.46) | 0.493 | 0.39 (0.05-2.79) | 0.344 | 0.47 (0.07-3.09) | 0.425 |
|  | COVID-19 severity (cat.) | **2.52 (1.24-5.11)** | **0.011** | 0.88 (0.42-1.83) | 0.727 | 1.28 (0.63-2.59) | 0.489 |

Statistically significant results are written in bold font.
