# Supplementary material for: SARS-CoV-2 specific immune responses in overweight and obese COVID-19 patients
Source: Front Immunol. 2023 Nov 2;14:1287388. doi: 10.3389/fimmu.2023.1287388 (PMC10653322; doi:10.3389/fimmu.2023.1287388)
Supplement: Supplementary file 4 [file Table_4.docx]

**Supplementary table 4**

Variables associated with frequencies of spike- and non-spike-specific total cytokine secreting T cells after SARS-CoV-2 infection

| **Time post infection (months):** | **Variables:** | **Spike total cytokines**  **SFU/10^6^ PBMC**  **Adjusted estimate (95% CI) p-value** | | | **Non-spike total cytokines**  **SFU/10^6^ PBMC**  **Adjusted estimate (95% CI) p-value** | | |
| --- | --- | --- | --- | --- | --- | --- | --- |
| **6** | BMI (cont.) | 1.11 (1.00-1.24) | 0.055 | 1.10 (0.99-1.23) | | 0.079 |  |
|  | Age (cont.) | 0.99 (0.96-1.01) | 0.297 | 1.00 (0.97-1.02) | | 0.811 |  |
|  | Gender (ref:male) | **2.15 (1.00-4.61)** | **0.050** | 1.74 (0.80-3.78) | | 0.156 |  |
|  | Any comorbidity (ref:no) | **3.09 (1.36-7.01)** | **0.008** | **2.35 (1.02-5.38)** | | **0.045** |  |
|  | COVID-19 severity (cat.) | 1.17 (0.89-1.55) | 0.263 | 1.09 (0.82-1.44) | | 0.550 |  |
| **12** | BMI (cont.) | **1.07 (1.01-1.14)** | **0.022** | 1.10 (0.97-1.25) | | 0.124 |  |
|  | Age (cont.) | **1.02 (1.00-1.03)** | **0.038** | 1.01 (0.97-1.05) | | 0.602 |  |
|  | Gender (ref:male) | 1.60 (0.97-2.65) | 0.067 | 2.58 (0.88-7.59) | | 0.084 |  |
|  | Any comorbidity (ref:no) | 1.25 (0.75-2.08) | 0.395 | 0.66 (0.22-1.97) | | 0.452 |  |
|  | COVID-19 severity (cat.) | 1.14 (0.95-1.39) | 0.162 | 1.32 (0.88-1.99) | | 0.178 |  |

Statistically significant results are written in bold font.
