# Supplementary material for: SARS-CoV-2 specific immune responses in overweight and obese COVID-19 patients
Source: Front Immunol. 2023 Nov 2;14:1287388. doi: 10.3389/fimmu.2023.1287388 (PMC10653322; doi:10.3389/fimmu.2023.1287388)
Supplement: Supplementary file 5 [file Table_5.docx]

**Supplementary table 5**

Details on COVID-19 vaccination at 18 months post infection

| **Vaccinated by 18 months**  n= 169 | **Normal weight**  n=84 | **Overweight**  n=62 | **Obese**  n=23 |
| --- | --- | --- | --- |
|  |  |  |  |
| One vaccine dose | 63 (75%) | 52 (84%) | 20 (87%) |
| Days since vaccination  Median (IQR) | 136 (108-165)* | 165 (121-196) | 183 (143-198) |
|  |  |  |  |
| Two vaccine doses | 21 (25%) | 10 (16%) | 3 (13%) |
| Days since vaccination  Median (IQR) | 97 (47-156) | 84 (14-169) | 56 (38-118) |

*Vaccination date was missing for one participant, IQR=interquartile range
