# Supplementary material for: SARS-CoV-2 specific immune responses in overweight and obese COVID-19 patients
Source: Front Immunol. 2023 Nov 2;14:1287388. doi: 10.3389/fimmu.2023.1287388 (PMC10653322; doi:10.3389/fimmu.2023.1287388)
Supplement: Supplementary file 6 [file Table_6.docx]

**Supplementary table 6**

Descriptive summary of results following SARS-CoV-2 infection and COVID-19 vaccination focusing on overweight and obese patients compared to normal weight patients

|  | **Normal weight** | **Overweight** | **Obese** |
| --- | --- | --- | --- |
| **Following SARS-CoV-2 infection** |  |  |  |
| Spike-specific IgG levels | + | ++^a^ | +++^b^ |
| Fold spike-IgG reduction | - | - -^c^ | - - |
| Neutralising antibody levels | + | ++^c^ | ++^d^ |
| Fold neutralising antibody reduction | - | - -^e^ | - - |
| Neutralising/spike-IgG ratio | ++ | ++ | + |
| IgA % positive samples (RBD) | + | ++ | ++ |
| IgM % positive samples (RBD) | + | ++ | ++ |
| TCR spike breadth (6 and 12 m) | + | ++ | +++^c^ |
| TCR spike depth (6 and 12 m) | + | ++ | +++^f^ |
| TCR non-spike breadth (6 and 12 m) | + | ++^c^ | +++^c^ |
| TCR non-spike spike depth (6 and 12 m) | + | ++ | +++^f^ |
| Spike-specific total T cells | + | ++^f^ | ++^f^ |
|  |  |  |  |
| **Following COVID-19 vaccination** |  |  |  |
| Spike-specific IgG levels | +++ | +++ | +++ |
| Neutralising antibody levels | +++ | +++ | +++ |
| Neutralising/spike-IgG ratio | ++ | ++ | + |
| Fold spike-IgG increase | ++ | + | + |
| Fold neutralising antibody increase | ++ | +^c^ | +^c^ |
| Spike-specific total T cells | ++ | + | + |
| Spike-specific MBCs | ++ | + | + |

^a^significant at 2, 4 and 6 months, ^b^significant at 2, 6 and 12 months, ^c^significant, , ^d^significant at 2 and 12 months, ^e^significant at 6 months, ^f^significant at 12 months,
