# Supplementary material for: SARS-CoV-2 specific immune responses in overweight and obese COVID-19 patients
Source: Front Immunol. 2023 Nov 2;14:1287388. doi: 10.3389/fimmu.2023.1287388 (PMC10653322; doi:10.3389/fimmu.2023.1287388)
Supplement: Supplementary file 7 [file DataSheet_1.pdf]

Supplementary Figure 1

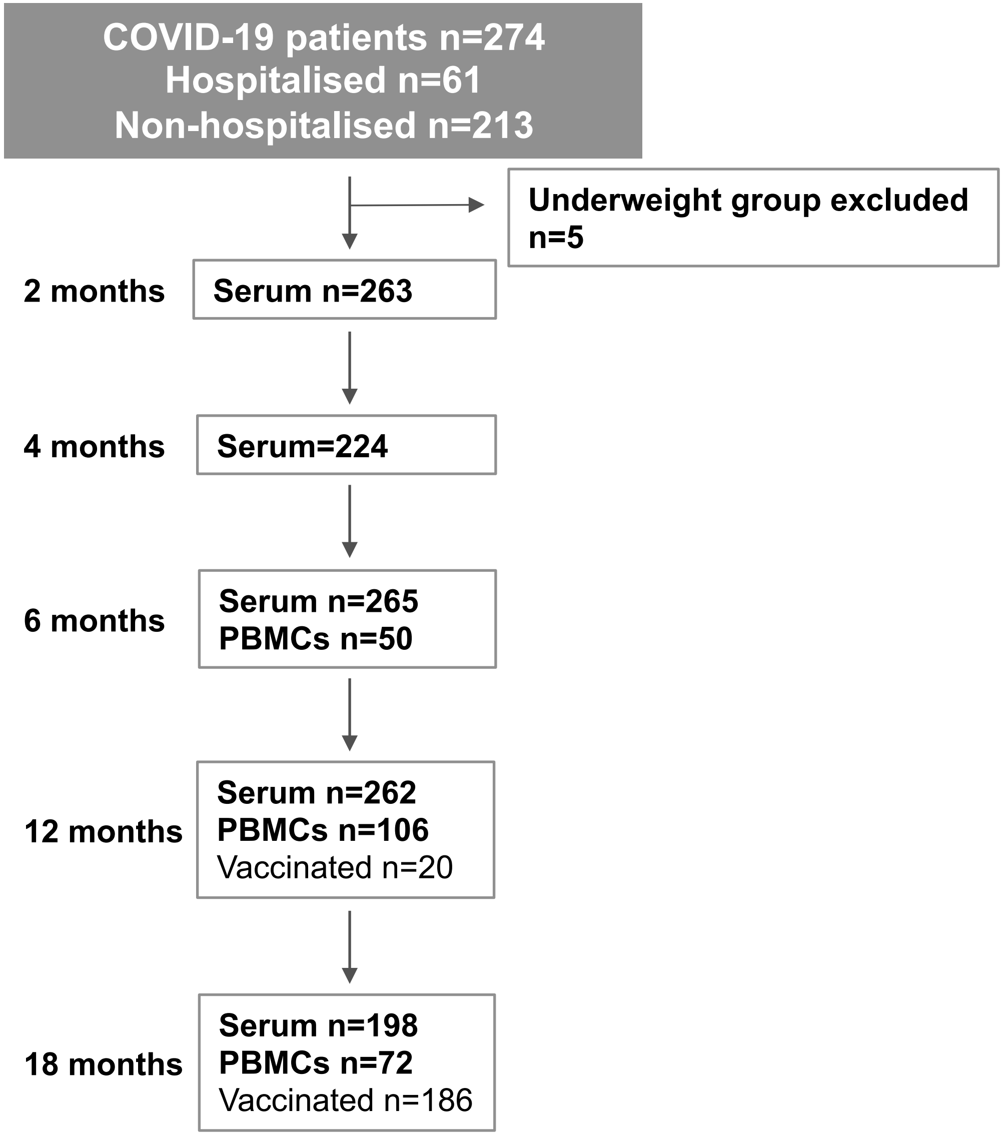

Supplementary Figure 1.
Overview of the numbers of participants included in the study, and that provided serum samples, PBMCs and that had been vaccinated against COVID-19 at the follow-up visits. Participants were SARS-CoV-2 RT-PCR positive and/or had positive serology at the time of inclusion.
