# Supplementary material for: SARS-CoV-2 specific immune responses in overweight and obese COVID-19 patients
Source: Front Immunol. 2023 Nov 2;14:1287388. doi: 10.3389/fimmu.2023.1287388 (PMC10653322; doi:10.3389/fimmu.2023.1287388)
Supplement: Supplementary file 8 [file DataSheet_2.pdf]

Supplementary Figure 2

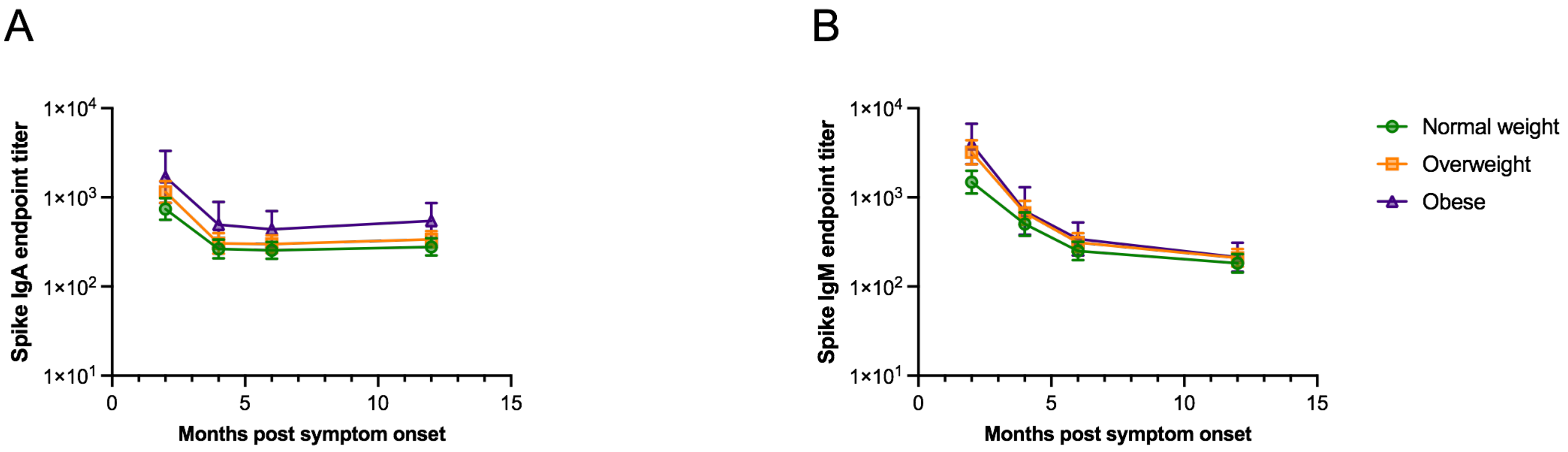

Supplementary Figure 2. Spike-specific IgA **(A)** and IgM **(B)** endpoint titres were determined by ELISA at 2, 4, 6 and 12 months post symptom onset for normal weight, overweight and obese patients.
